# TRP Channels as Sensors of Aldehyde and Oxidative Stress

**DOI:** 10.3390/biom11101401

**Published:** 2021-09-24

**Authors:** Katharina E. M. Hellenthal, Laura Brabenec, Eric R. Gross, Nana-Maria Wagner

**Affiliations:** 1 Department of Anesthesiology, Intensive Care and Pain Medicine, University Hospital Muenster, 48149 Muenster, Germany; katharina.hellenthal@uni-muenster.de (K.E.M.H.); laura.brabenec@ukmuenster.de (L.B.); 2 Department of Anesthesiology, Perioperative and Pain Medicine, Stanford University, Stanford, CA 94305, USA; ergross@stanford.edu

**Keywords:** aldehyde, TRP channels, ALDH2, oxidative stress, TRPV1

## Abstract

The transient receptor potential (TRP) cation channel superfamily comprises more than 50 channels that play crucial roles in physiological processes. TRP channels are responsive to several exogenous and endogenous biomolecules, with aldehydes emerging as a TRP channel trigger contributing to a cellular cascade that can lead to disease pathophysiology. The body is not only exposed to exogenous aldehydes via tobacco products or alcoholic beverages, but also to endogenous aldehydes triggered by lipid peroxidation. In response to lipid peroxidation from inflammation or organ injury, polyunsaturated fatty acids undergo lipid peroxidation to aldehydes, such as 4-hydroxynonenal. Reactive aldehydes activate TRP channels via aldehyde-induced protein adducts, leading to the release of pro-inflammatory mediators driving the pathophysiology caused by cellular injury, including inflammatory pain and organ reperfusion injury. Recent studies have outlined how aldehyde dehydrogenase 2 protects against aldehyde toxicity through the clearance of toxic aldehydes, indicating that targeting the endogenous aldehyde metabolism may represent a novel treatment strategy. An addition approach can involve targeting specific TRP channel regions to limit the triggering of a cellular cascade induced by aldehydes. In this review, we provide a comprehensive summary of aldehydes, TRP channels, and their interactions, as well as their role in pathological conditions and the different therapeutical treatment options.

## 1. Introduction

Exposure to endogenous and exogenous aldehydes has emerged as a key contributor to disease pathology in recent years [1,2]. The body is exposed to exogenous reactive aldehydes through such things as tobacco products or alcoholic beverages [2,3,4]. Importantly, aldehydes are also an endogenous product derived from the peroxidation of polyunsaturated fatty acids during oxidative stress and are elevated in acute disease states, such as myocardial reperfusion injury or sepsis, and chronic disease states—for example, diabetes, cancer, or neurodegeneration [5,6]. Reactive aldehydes mediate detrimental actions on the body and exposure significantly impairs human health [7,8] by exerting direct cytotoxicity, causing DNA damage and the induction of cell death. In the past few years, it has become clear that reactive aldehydes also trigger conformational changes in the transient receptor potential (TRP) channel, inducing a pro-inflammatory cascade. 

TRP cation channels were originally known for their essential role in nociception, but recent evidence suggests their involvement in a multitude of pathophysiological conditions; TRP channels thus contribute far more to cellular process beyond merely nociception [9,10,11]. Recently, evidence that aldehydes form adducts with TRP channels, leading to the activation of the cation channel and the subsequent calcium-induced release of pro-inflammatory mediators, has been accumulating [12]. Thus, TRP channels act as biosensors of reactive aldehydes and oxidative stress. Recent evidence demonstrates that reactive aldehydes themselves and through interaction with the TRP channel induce endothelial dysfunction in acute disease states, such as sepsis, and chronic disease states, such as diabetes [13,14,15,16,17]. As endothelial dysfunction is a key characteristic of the pathophysiology of multiple diseases, these findings provide a novel baseline for pharmacological treatment aiming for the preservation of vascular integrity. 

This review focuses on the formation of endogenous aldehydes; their precise interaction with TRP cation channels—especially the TRPV1 and TRPA1 channels; and aims to dissect the possibilities for pharmacological strategies targeting their interface. 

## 2. Reactive Oxygen and Reactive Aldehydes as Signaling Molecules

Oxidative stress occurs during an imbalance between antioxidant regulations and the production of reactive oxygen species (ROS) and has been identified as a key driver of pathologies—for example, in diabetes, neurogenic inflammation, cancer, neurodegenerative diseases, and sepsis [7,18]. ROS include chemical species with an unpaired electron—for instance, hydroperoxyl radical (HO•_2_), superoxide radical (O_2_ •−), or hydroxyl radical (•OH), which are formed as normal by-products of cellular metabolism and play important roles in physiological processes. For instance, ROS essentially contribute to immune defense against invading pathogens via an oxidative burst in phagocytes [19]. Further, the mitochondrial electron transport chain physiologically produces ROS through oxidative phosphorylation, resulting in the production of ATP from the reduction of molecular oxygen (O_2_) to water (H_2_O) [20]. During aerobic cell metabolism, different mitochondrial complexes constantly release superoxide radicals (O_2_ •−) into the mitochondrial matrix, which are further converted to hydrogen peroxide (H_2_O_2_) [21]. Mitochondrial aconitase mediates the formation of hydroxyl radicals (•OH) from H_2_O_2_ via the Fenton reaction [22]. Besides the mitochondrial enzyme transport chain, the cytochrome P450 (CYP) enzymes metabolizing organic substrates are key sources of endogenous ROS formation in mammals. CYP enzymes are expressed in the membranes of mitochondria and the endoplasmic reticulum and catalyze the oxygenation of multiple substrates and the simultaneous reduction of molecular oxygen, leading to the formation of ROS [23,24]. 

During states of inflammation, ischemia, infection, or exposure to environmental irritants, an imbalance of ROS exists, causing tissue injury [25]. The unpaired electron of ROS leads to an attack on macromolecules, further to damage to the DNA, the oxidation of amino acids in proteins, the deactivation of enzymes, the formation of further radicals, and thus an amplification of the response to oxidative stress [26]. 

An example illustrating the specific role of oxidative stress in inflammation is how ROS substantially affect vascular barrier function in systemic inflammation during sepsis. Vascular leakage upon systemic pathogen infiltration has been known to be a major contributor to sepsis mortality, as it leads to impaired tissue perfusion and oxygen supply to cells and thus to organ dysfunction and septic shock [27,28]. The excessive activation of the endothelium leads to fluid shifting into the extravascular space and is thus a key contributor to the induction of vascular leakage [29]. During sepsis, endothelial cells are both a target and a source of ROS [18]. PAMPs (pathogen-associated molecular patterns) and DAMPs (damage-associated molecular patterns) of the emerging pathogen induce endothelial ROS production, primarily in the mitochondria, which further initiates a cascade of events causing a sepsis phenotype [18,30]. ROS lead to a procoagulant, proadhesive, and pro-inflammatory endothelial cell state; promote capillary leakage; and alter vasomotor tone [31]. ROS are thus crucial products and mediators of systemic inflammation and key contributors to sepsis complications, such as organ dysfunction.

ROS also trigger the release of reactive aldehydes through lipid peroxidation, which substantially amplifies the oxidative stress cascade. Polyunsaturated acids, such as arachidonic or linoleic acid, and polyunsaturated acyl chains of phospholipids in membranes are highly susceptible to attack by radicals, as their double bond leads to a weak carbon–hydrogen bond. The peroxidation of n-3 and n-6 fatty acids, which are essential fatty acids characterized by a double bond between the second and third (n-3) or sixth and seventh (n-6) carbon atoms from the terminal methyl group, by ROS leads to the nonenzymatic generation of endogenous reactive aldehydes, such as 4-hydroxy-2E-hexenal (4-HHE), malondialdehyde (MDA), and 4-hydroxy-2E-nonenal (4-HNE) [32]. 

When compared to reactive free radicals, reactive aldehydes exert prolonged activity, migrate throughout the whole body, and can thus reach sites distant from the initiating free radical event and can subsequently act as a “second toxic messenger” in the oxidative stress cascade [33]. Further, they amplify the reactive oxygen response itself as lipid radicals (L•) and react with oxygen, forming a peroxyl radical (LOO•) that further generates a new lipid radical (L•) and lipid hydroperoxide (LOOH) by the separation of a hydrogen from another lipid molecule [34].

Reactive aldehydes are not only products of oxidative stress, but also—and more importantly—mediators of oxidative stress. The impact of 4-HNE on the human body has been extensively studied in the past few years. 4-HNE has a specific reactivity profile as it contains three main functional groups: a C1=O carbonyl group, a C=C double bond at C2, and a further hydroxy group at C4 mediating reactions [6]. Upon the induction of oxidative stress, the intracellular levels of 4-HNE increase up to 100-fold and, similarly to ROS, 4-HNE exerts cytotoxic and cancerogenic effects. 4-HNE forms Michael-type adducts via its carbon double bond with cysteine, lysine, and histidine residues, leading to functional changes in proteins—for example, in enzymes or ion channels [35]. Further, 4-HNE impairs the function of cellular transporters such as Na^+^/K^+^-ATPase or GLUT 3 [1,36]. 4-HNE modulates the inflammatory response by the induction of cyclooxygenase-2 (COX-2) and TNF-α [35]. 4-HNE induces intrinsic apoptosis and necrosis and leads to the depletion of glutathione (GSH), a key regulator in the detoxification of cytotoxic material [9,10]. Moreover, 4-HNE exerts mutagenic effects by the modification of DNA [37,38]. Additionally, the excessive peroxidation of the lipid membrane itself leads to increasing permeability, decreasing membrane potential, and the potential rupture of the cell membrane [26]. 

In the past few years, it has become clear that reactive aldehydes also specifically modify vascular function. Low physiological concentrations of 4-HNE appear to be vasoprotective and positively affect endothelial cell proliferation and the cell cycle [13]. However, similar to ROS, high levels of aldehydes significantly impair vascular function and are thus contributors to disease pathophysiologies—for example, this can occur during sepsis [13,14]. Even decades ago, studies demonstrated that the infusion of 4-HNE leads to the formation of lung edema in rodents and induces albumin extravasation through an endothelial monolayer [15,16]. In their studies, Usatyuk et al. illustrated the molecular mechanism underlying the edema-inducing effects by showing that 4-HNE disrupts the endothelial barrier by affecting the MAPK signaling cascade, resulting in the remodeling of the actin cytoskeleton [39]. 

**Figure 1:**
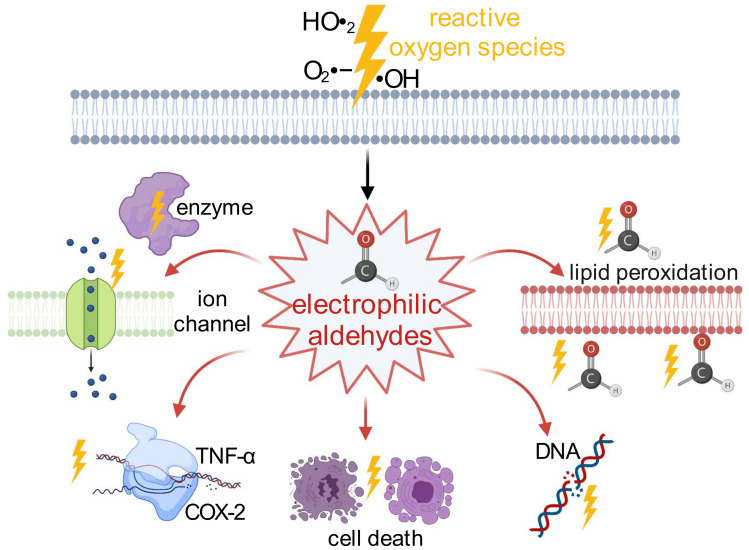
Production of endogenous reactive aldehydes by reactive oxygen species and their adverse actions on the body. During oxidative stress, reactive oxygen species, such as hydroperoxyl radical (HO•_2_), superoxide radical (O_2_ •−), and hydroxyl radical (•OH), induce the peroxidation of polyunsaturated acids, leading to the release of electrophilic aldehydes such as 4-hydroxy-2E-hexenal (4-HHE), malondialdehyde (MDA) and 4-hydroxy-2E-nonenal (4-HNE). These reactive aldehydes further induce conformational changes in enzymes and ion channels; the production of pro-inflammatory substrates such as tumor necrosis factor α (TNF-α) and cyclooxygenase-2; cellular necrosis and apoptosis; DNA damage; the lipid peroxidation of membranes; and the amplification of the cascade by the production of new electrophilic aldehydes. This figure was created with BioRender.com (accessed on 4 September 2021).

In light of these findings, it is evident that reactive aldehydes are far more than just a byproduct of oxidative stress, as they exert systemic cytotoxic and mutagenic potential and cause detrimental effects even at sites distant from the initial free radical event (Figure 1). Further, reactive aldehydes may display a novel target in diseases driven by oxidative stress to escape the vicious cycles of injury they are accounting for. In the past few years, several studies have suggested that TRP channels may also be a target of reactive aldehydes. 

## 3. From a Spontaneous Mutation to Its Own Research Field: TRP Channels in History 

TRP channels have recently become the focus of research as a potential target for reactive aldehydes. As mentioned above, reactive aldehydes and oxidative stress play key roles in various pathological conditions, and their interaction with TRP channels is therefore of great interest for therapeutic options. Originally, TRP channels were identified as receptors for environmental perception and nociception. The history of TRP channels and their discovery and structure, which are important factors for aldehyde interaction, are briefly described in the following section.

In 1969, after the discovery of a transient receptor potential response in *Drosophila* exposed to constant brightness following a spontaneous mutation, the attention of researchers was drawn to the *trp* (transient receptor potential) gene [40]. It took another 16 years for Craig Montell, an American scientist, to identify and clone a DNA fragment responsible for the defect and restoration of the visual sense of *Drosophila* through the identification of the first *trp* gene [41]. Later, he introduced a unified nomenclature for the TRP superfamily after TRP ion channels came more and more to be the focus of researchers. Since then, TRP channels have been widely discovered and characterized due to their genomic similarity (TRP channels are extensively reviewed in [10,11]).

**Figure 2:**
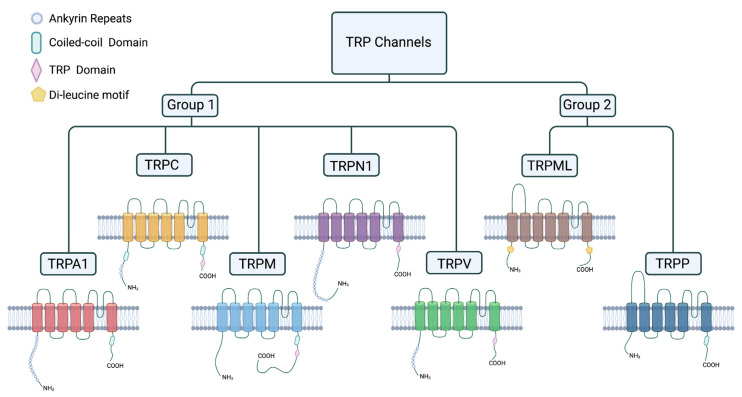
TRP channels and their structure. TRP channels consist of six transmembrane segments and can be divided into group 1 and group 2 cation channels depending on their structure and amino acid sequence. Group two TRP channels have a larger extracellular loop between the first and the second transmembrane segment. This figure was created with BioRender.com (accessed on 4 September 2021).

The metazoan cation channel superfamily can be divided into two subfamilies: the group 1 subfamily, comprising the TRPA (ankyrin), TRPC (canonical), TRPM (melastatin), TRPN (nompc), and TRPV (vanilloid) cation channels, and the group 2 subfamily, comprising the TRPP (polycystic) and TRPML (mucolipin) channels [10,11]. The TRP channels consist of six transmembrane segments, with the N- and C-termini being directed toward the cytoplasm and differing in their length and domains. The group 1 members TRPA, TRPC, TRPN, and TRPV have variable ankyrin-repeats at the N-terminal end, whereas some but not all TRPM channels have a large kinase domain at the C-terminal end (Figure 2). Compared to group 2, members of group 1 have a strong overlap with the first identified *Drosophila trp* gene. The group 2 members TRPP and TRPML have less homology with the first identified *trp* gene but are not the focus of this review [10,11].

The TRPC subfamily is divided into a total of seven different TRPC cation channels (TRPC1, TRPC2, TRPC3, TRPC4, TRPC5, TRPC6, TRPC7), which are either canonical or classical TRP channels. The first mammalian TRPC ion channel TRPC1 was cloned by Wes et al. in 1995 [42]. They are permeable for Ca^2+^ with different selectivities and phospholipase C is needed for activation [10,11]. 

The subtype TRPA1 cation channel is named after the major ankyrin repeat domain at the N-terminal end of the receptor. It is mainly expressed on primary afferent nociceptors positive for TRPV1 and other cell types. TRPA1 is permeable to Ca^2+^, Na^+^, and K^+^ flux. The permeability to those cations can cause a discharge in action potential, membrane depolarization, and neurotransmitter release. TRPA1 was first discovered in 2003 by Story et al. [43] and named ANKTM1; it was first identified to be involved in the sensation of cold. Later, it was discovered that TRPA1 is also stimulated and activated by itching; pain; and different pungent compounds of garlic, cinnamon, and wasabi—for example, allicin and allyl isothiocyanate or cinnamaldehyde [11,44,45,46].

The vanilloid-related TRP channel (TRPV) family consists of a total of six subtypes: TRPV1, TRPV2, TRPV3, TRPV4, TRPV5, and TRPV6. The first member of the TRPV subfamily, the nonselective cation channel TRPV1, was discovered in 1997 by Caterina et al. and found to be a receptor addressed by the vanilloid-like molecule capsaicin [47]. Five more TRPV channels have been discovered since [11,48,49,50,51,52,53]. Both TRPV5 and TRPV6 are involved in calcium homeostasis, since they have a very high calcium selectivity compared to other TRP channels. TRPV cation channels are expressed on sensory nociceptors and non-neuronal tissues and can be activated by a variety of stimuli, such as heat, capsaicin, and toxins. TRPV1 is a cation channel that is activated by heat, capsaicin, low pH (H^+^), and leukotriene B4, resulting in a calcium (Ca^2+^) influx. TRPV2, on the other hand, is activated by higher temperatures but, similar to TRPV3 and TRPV4, it is not activated by capsaicin or an acidic environment. Both TRPV3 and TRPV4 are activated by moderate temperatures. TRPV5 and TRPV6, as channels highly selective for calcium, are mainly expressed in organs that are important for calcium homeostasis [54]. 

The first of the eight melastatin TRP members of the TRPM subfamily was identified by Duncan et al. in 1998 [55]. TRPM2, 6, and 7 not only function as permeable cation channels, but also have an enzymatic functionable domain at their C-terminus [10,11]. 

TRPN1 (NompC) is a channel that is important for mechanosensation and mostly occurs in worms, zebrafish, and flies, but not in mammals. With 28–29 ankyrin repeats, it has more ankyrin repeats at the N-terminal end than any other TRP cation channel [10]. 

In summary, the TRP superfamily consists of many subfamilies and offers versatile therapeutic targets, as the families can be stimulated by a variety of different stimuli, such as aldehydes.

## 4. Reactive Aldehydes and TRP Channels: A Critical Interface in Disease Pathophysiology

In recent years, the interaction between reactive aldehydes and TRP channels has become more recognized. Reactive aldehydes are the peroxidation products of n-3 and n-6 fatty acids in membranes derived from two different pathways: an enzymatic reaction requiring 15-lipoxygenase (15LOX) or 12-lipooxygenase (12LOX) and non-enzymatic generation by reactive oxygen species during oxidative stress [32]. Both pathways produce reactive aldehydes that specifically interact with TRP channels. This section deals with the crosstalk of aldehydes and the two major TRP channels involved in diseases: the transient receptor vanilloid 1 and the ankyrin 1 channel (TRPV1 and TRPA1, respectively). We will pay special attention to 4-hydroxy-2E-nonenal (4-HNE), as this is one of the most extensively studied aldehydes and affects the function of both channels. In the following, we will provide an overview of the specific interaction sites and consequences resulting from TRP channels being sensors of aldehydes during oxidative stress. 

TRP channels function as biosensors for eicosanoids derived from phospholipid membranes. For instance, lysophosphatidic acid (LPA) and phosphatidylinositol 4,5-biphosphate (PIP_2_) activate the TRPV1 channel via the modification of lysine 710 (K710) at its C-terminal region [56,57]. Further, recent evidence from our research group has shown that the eicosanoid 12(S)-hydroxyeicosatetraenoic acid (12(S)-HETE) specifically interacts with the TRPV1 channel, leading to diabetes-induced vascular complications. Hyperglycemia in diabetes leads to an increase in the 12(S)-hydroxyeicosatetraenoic acid (12(S)-HETE) plasma levels due to the enzymatic breakdown of polyunsaturated acids catalyzed by 12LOX [58]. As 12(S)-HETE has structural similarities with LPA and PIP_2_, it binds to intracellularly located TRPV1 receptors and leads to the promotion of endothelial dysfunction in diabetes by triggering mitochondrial calcium influx and subsequent mitochondrial dysfunction [59,60]. Individuals with diabetes exhibit impaired endothelial function due to calcium overload in the mitochondria, resulting in a loss of membrane potential and the impairment of the mitochondrial respiration chain, making mitochondria unable to produce the energy currency of the cell ATP [61,62]. Further, mitochondrial calcium overload induces a release of cytochrome c, which initiates the apoptosis of the cell [63,64]. Importantly, defect mitochondrial calcium homeostasis reduces the bioavailability of nitric oxide, which leads to defective flow-mediated vasodilatation and thus to vascular abnormalities, promoting diabetes complications [65,66,67]. Interestingly, mitochondrial calcium influx induces the release of ROS, which in turn triggers calcium influx into the mitochondria, creating a vicious circle [68].

The crosstalk between 12(S)-HETE and the TRPV1 receptor provides a possible link between elevated glucose levels in diabetes leading to mitochondrial calcium overload and related vascular complications—for example, myocardial infarction or stroke—and, subsequently, is a novel therapeutic target. The measurement of the lipid peroxidation product concentration may further help in risk stratification for adverse cardiovascular events in diabetic patients. 

Conversely, as mentioned earlier in this review, oxidative stress triggers the nonenzymatic breakdown of reactive aldehydes, which in turn leads to functional changes in proteins by the covalent modification of lysine (ε-amino group), histidine (imidazole group), or cysteine residues (sulfhydryl group) [69]. Interestingly, the modification of TRP channels by endogenous aldehydes differs from activation by classic agonists, such as capsaicin, which directly binds to TRP channels by classic lock-and-key binding, as these substances lack a reactive group [70]. In the following, we will discuss how endogenous aldehydes interact with the TRPV1, TRPA1, and TRPC channels. 

In their studies, DelloStritto et al. showed that the lipid peroxidation product 4-hydroxy-2E-nonenal (4-HNE) leads to the impaired function of the TRPV1 channel due to the modification of the cysteine residue C621 located at the key pore helices of the channel [71]. They analyzed the binding of 4-HNE to the TRPV1 channel using human embryonic kidney 293 (HEK293) cells transfected with wild type and a C621G mutant of the TRPV1 channel. In immunoblot quantification, they observed increased the interaction between WT-TRPV1 and 4-HNE compared to that between vehicle cells, whereas 4-HNE interaction with TRPV1 was decreased in cells expressing a TRPV1 mutant, in which the crucial cysteine residue was replaced by a glycine amino acid residue. Further, the C621G mutation rescued the 4-HNE-mediated decrease in the capsaicin-dependent current in a patch clamp analysis. These results point towards the direct posttranslational modification of cysteine residue C621 by 4-HNE in the pore-forming region, causing a decrease in the TRPV1 channel activity and leading to the impairment of vascular physiology. The TRPV1 channel physiologically integrates hemodynamic responses and vasoreactivity, whereas diminished TRPV1 function in turn leads to vascular dysfunction [17]. As the levels of 4-HNE are increased in diabetes, DelloStritto et al. provided a link between diabetes and cardiovascular disease due to the impairment of the TRPV1 channel activity via the posttranslational modification of cysteine residue C621 by 4-HNE [5,71]. Interestingly, earlier studies from their group have elucidated that the ROS H_2_O_2_ similarly affects TRPV1 channel activity by showing that TRPV1 function is decreased by prolonged exposure to H_2_O_2_ [72]. TRPV1 was shown to induce nitric oxide production in earlier studies and negatively affect TRPV1 channel function. [73]. As 4-HNE and H_2_O_2_ impair TRPV1 function, they may further lead to a deficiency in nitric oxide production, a central characteristic of diabetes-induced endothelial dysfunction, as described earlier in this review, when illustrating 12(S)-HETE) and TRPV1 interaction [65,66,67].

Besides 4-HNE targeting the TRPV1 channel, there has been some evidence pointing towards the notion that TRPV1 is activated by other reactive aldehydes. 5,6-epoxyeicosatrienoic acid, a metabolite derived from arachidonic acids, leads to the opening of the TRPV1 channel, and there has been some evidence that the oxidized linoleic acid metabolites 9- and 13-hydroxyoctadecadienoic acid (9-HODE, 13-HODE), which are induced by noxious heat, activate TRPV1 and thus contribute to heat sensitivity in rodents [74,75]. Patwardhan et al. showed that the heat sensitivity of TRPV1 is regulated by the generation of linoleic acid metabolites both in vitro and in vivo. First, they discovered that heated skin evokes an increase in the intracellular calcium levels, whereas heat administered to TRPV1 knockout mice did not induce calcium changes nor lead to nociceptive behavior in these mice. Next, they showed that 9-HODE as well as 13-HODE are released upon heat exposure in mice and that both aldehydes induce calcium inward currents in cells transfected with TRPV1. Interestingly, the pretreatment of AMG8562, which is a highly potent blocker of capsaicin action on TRPV1 that is unable to block the heat activation of TRPV1, did not affect the actions of 9-HODE and 13-HODE on TRPV1, suggesting that the interaction site may differ from the classic capsaicin binding site. The infusion of antibodies against HODEs into the ipsilateral hind paw of rodents substantially reduced their nociceptive response to heat, whereas heat responsiveness was preserved in the contralateral paw with the injection of the vehicle [75]. These findings point towards the notion that TRPV1 channels sense aldehydes and thus mediate responsiveness to noxious heat. TRPV1 channels thus function as potent biosensors integrating the stimuli of reactive aldehydes during physiological and pathophysiological states. 

**Figure 3:**
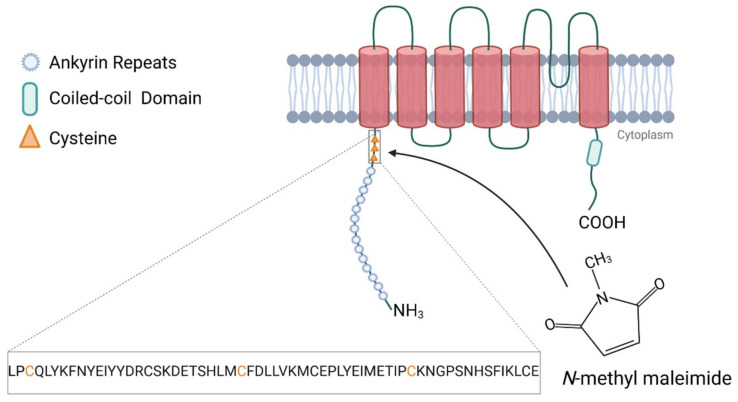
TRPA1 ion channel and N-terminal cytoplasmic interaction site with *N*-methyl maleimide. Cysteine residues necessary for NMM interaction are illustrated in orange [76]. Created with BioRender.com (accessed on 4 September 2021).

When shedding light on the interface between TRPA1 channels and reactive aldehydes, there is a large body of evidence illustrating their sites of interactions. As the structures of natural TRPA1 agonists significantly differ, Hinman et al. hypothesized that the reactivity of the agonists rather than the structure itself is crucial for activation by reactive aldehydes [76]. Their key findings reveal that the electrophilic agonists of TRPA1 lead to the covalent modification of cysteine residues C619, C639, and C663 and part of lysine residue K708 at the cytoplasmatic N-terminal end of the channel. The substitution of the three cysteine residues abolished the actions of 2-aminoethyl methanethiosulfonate hydrobromide (MTSEA) and *N*-methyl maleimide (NMM), two highly reactive chemicals, on TRPA1 channels (Figure 3). 

Interestingly, the administration of 2-trimethylammoniumethyl methanethiosulfonate (MTSET), a membrane-impermeable variant, did not affect TRPA1 function, indicating that the cytoplasmatic site of TRPA1 channel is crucial for channel activation. Conversely, allyl isothiocyanate (AITC) did still induce a slow response at the TRPA1 triple cysteine mutant, but the irreversible modification of the lysine-708 residue completely abolished activation by AITC, suggesting that lysine residue may also be a site of covalent modification by electrophilic TRPA1 agonists. Replacing the three cysteine residues does not impact the sensitivity of TRPA1 to δ-9-tetrahydrocannabinol (THC) or 2-aminophenyl borane (2-APB), which do not exert electrophilic actions, suggesting that electrophilicity is required for the covalent modification of C619, C639, and C663. The N-terminal site of the TRPA1 channel may thus play a crucial role in regulating channel activity and act as a biosensor for noxious electrophilic agonists mediating conformational changes [76].

Later studies have confirmed that electrophilic compounds affect specific N-terminal cysteine residues of the TRPA1 channel by in vivo labeling and mass spectrometry. The induction of the disulfide interactions of these cysteine residues is suggestive of leading to conformational changes and thus to the activation of the channel [77]. 

Conversely, it has been shown that other regions of the TRPA1 channel may also contribute to the response to stimulation by reactive electrophiles, as in absence of the N-terminal region the channel activation by electrophilic compounds was still maintained [78]. Subsequently, further interaction sites between reactive chemicals and TRPA1 channels that will need to be elucidated in future investigations may exist.

In recent years, interaction between the endogenous reactive aldehyde 4-hydroxy-2E-nonenal (4-HNE), which is produced upon exposure to oxidative stress, as described above, and the TRPA1 channel has gained much attention. 

In their studies, Trevisani et al. addressed this issue and showed that 4-HNE dose-dependently activates the TRPA1 channel by the covalent modification of cysteine residues, as a genetically modified TRPA1 channel triple mutant (TRPA1-3C) in oocytes lacking these specific cysteine residues was protected against the effects of 4-HNE. First, they showed that 4-HNE elicits a dose-dependent increase in intracellular calcium concentrations in HEK293 cells expressing the TRPA1 channel, whereas vector-transfected control cells did not respond to the 4-HNE treatment. Suggesting that 4-HNE may directly affect TRPA1 channel function, they also determined HNE-evoked responses using calcium imaging in cultured sensory neurons and similarly observed responsiveness to 4-HNE. Conversely, TRPA1-deficient neurons did not show any response to 4-HNE treatment. After identification that 4-HNE may affect TRPA1 activity, they gained a closer insight into the concise interaction sites. Using voltage clamp recording during 4-HNE exposure, they detected the activation of the TRPA1 channel, whereas the response to 4-HNE was significantly diminished in a mutant (TRPA1-3C). Furthermore, the TRPA1 antagonist camphor was found to protect mice against pain induced by 4-HNE, indicating that 4-HNE exerts pro-algesic actions via the activation of the TRPA1 channel [79]. Thus, the study by Trevisani et al. reveals that 4-HNE not only impairs TRPV1 function, as described earlier in this review, but also directly affects TRPA1 function.

Similarly, TRPA1 is activated by the exogenous reactive α,β-unsaturated aldehyde, acrolein [44]. Acrolein can be found in air pollution, tear gas, tobacco products, and as a toxic byproduct of chemotherapy. Acrolein exposure is closely linked to neurogenic inflammation in diseases such as asthma or chronic obstructive pulmonary disease [2,44,80]. Interestingly, the mainly exogenous-derived aldehyde acrolein also forms Michael adducts with cysteine, histidine, and lysine amino acids such as endogenous aldehydes [81].

The spectrum of endogenous aldehydes activating the TRPA1 channel was broadened by showing that the lipid peroxidation products 4-oxo-2-nonenal (4- ONE) and 4-hydroxyhexenal (4-HHE) trigger the calcium release of the TRPA1 channel [82]. Andersson et al. treated Chinese hamster ovary (CHO) cells expressing the TRPA1 channel with 4-HNE, 4-ONE, and 4-HHE and observed a calcium response from all three agonists. Interestingly, 4-HNE and 4-HHE had equipotent EC_50_ values, whereas the action of 4-ONE was more potent. These observations are in line with earlier studies, where 4-ONE was shown to be more neurotoxic and more protein-reactive, reflecting its greater thiol reactivity characteristics [83]. Importantly, the responses to 4-HNE, 4-HHE, and 4-ONE were significantly diminished in the dorsal root ganglia of TRPA1 knockout mice. Further, the reactive oxygen species hydrogen peroxide (H_2_O_2_) and 15- deoxy- Δ^12,14^-prostaglandin J2 (15d-PGJ2) and other reactive chemicals mediating tissue damage at high concentrations induced the activation of TRPA1. Conversely, TRPA1-deficient mice were protected against the actions of these products of oxidative stress. Interestingly, the administration of dithiothreitol (DTT), which reduces disulfide bonds, reversed the actions of H_2_O_2_ on the TRPA1 channel but not the actions of either 4-HNE or 15d-PGJ2, suggesting that H_2_O_2_ acts via the formation of disulfide bonds between vicinal cysteine residues, whereas the other reactive chemicals may form Michael adducts, as proposed earlier by Trevisani et al. [79,82].

In the context of the TRPA1 channel and its interaction with aldehydes, the TRPA1 response plays a key role in myocardial reperfusion injury. TRPA1 is not only expressed in pain sensory fibers, but also in smooth muscle cells, endothelial cells, and cardiomyocytes [84,85]. There has been evidence pointing towards acrolein not only being an exogenous-sourced reactive aldehyde, but also a direct lipid peroxidation product [86,87]. TRPA1 channel exposure to acrolein mediates myocardial-reperfusion injury in the setting of oxidative stress. Acrolein led to TRPA1-dependent intracellular calcium overload inducing myocardial cell death, whereas genetically modified TRPA1-deficient mice were significantly protected against myocardial ischemia-reperfusion injury [88]. In a mouse model of myocardial reperfusion injury caused by coronary artery occlusion and subsequent reperfusion, TRPA1 knockout mice exhibited a significantly reduced infarct size when compared to their wild-type littermates, suggesting the direct role of TRPA1 in myocardial reperfusion injury. As cardiac injury leads to the generation of lipid peroxidation products such as acrolein, Conklin et al. assessed the response of TRPA1 to acrolein in isolated cardiomyocytes. Exposure to acrolein resulted in an intracellular calcium increase, whereas calcium response was diminished by the administration of TRPA1 antagonist. The administration of acrolein to cardiomyocytes further induced hypercontracture by calcium overload, providing a direct link to TRPA1 channels being sensors of acrolein in the setting of cardiac infarction [88]. The induction of calcium overload is also a trigger of myocyte necrosis and arrhythmia, which points towards another pathophysiological role of acrolein-induced TRPA1-dependent calcium influx into cells during myocardial reperfusion injury. As other endogenous reactive aldehydes also induce the activation of TRP channels, as mentioned earlier, one can suggest that they may also mediate detrimental actions on cardiomyocytes and other cells. 

Shedding light on TRP channels other than TRPV1 and TRPA1 being affected by aldehyde exposure, a recent study determined that reduced thioredoxin leads to the activation of the TRPC5-TRPC1 channel [89]. Reduced thioredoxin, which is a modulator of oxidative stress, leads to a reduction of disulfide bonds in proteins and may thus lead to conformational changes in TRPC5-TRPC1 [90,91]. Similarly, cysteines Cys553 and Cys558 are nitrosylation sites of the TRPC5 channel, with the free radical nitric oxide mediating the activation of the channel [92]. These findings suggest that the covalent modification of TRPC channels during oxidative stress may also impair cellular function. 

It is important to take a closer look at the consequences of endogenous aldehyde and TRP interaction, as this provides a baseline for understanding why their interface substantially promotes the pathophysiology of diseases. When triggered by endogenous reactive aldehydes, the activation of TRPA1 as well as the TRPV1 channel leads to calcium overload into cells, which is a key trigger of cell death. Calcium influx into cells further induces the release of the neuropeptide substance P and calcitonin-gene related peptide (CGRP), which contribute to nociceptive hypersensitivity, providing a link to the role of aldehydes and further oxidative stress in neuropathic pain. For example, Trevisiani et al. showed that 4-HNE stimulates the release of CGRP and substance P from the spinal cord in rodents [79]. Further, the release of neuropeptides induces plasma extravasation, edema formation, and the infiltration of immune cells, all key mechanisms in the promotion of inflammation [93]. In line with this knowledge, 4-HNE was found to be a potent inducer of dose-dependent plasma extravasation, as measured by extravasated Evan’s blue content, which is a dye binding to albumin, in the hind paws of rats [79]. Importantly, the effects of aldehyde on TRP channels lead to the impairment of vascular function and vasoreactivity and may thus substantially contribute to disease pathophysiologies—for example, in sepsis during acute vascular dysfunction and in diabetes during a chronic state of vascular dysfunction. 

Thus, the interaction of endogenous aldehydes with TRP channels leads to a pro-algesic and pro-inflammatory state. Since reactive aldehydes are also elevated in chronic diseases such as diabetes, neurodegenerative disease, or rheumatoid arthritis [69], their interaction with TRP channels may also play a pivotal role in the promotion of chronic inflammation. Strikingly, reactive aldehydes are relatively long-lived and can travel through the body and thus potentially promote ubiquitous inflammatory responses, even at sites distinct from initial production [94]. Importantly, the interaction with the TRP channel leads to stable modification, indicating a prolonged response [95] and further suggesting that aldehydes may influence TRP channels in both short- and long-term responses.

Considering this large body of evidence showing that TRP channels display aldehyde sensors in the context of oxidative stress-induced tissue injury, this opens a new chapter of identifying specific interaction sites. Furthermore, their crosstalk may display a novel target in the treatment of multiple diseases. 

## 5. Targeting TRP Channels: A Multitude of Possibilities

TRP channels play a key role in acute and chronic diseases and targeting TRP channels—and especially their interaction with reactive aldehydes—may thus usher in a new era of treatment options. The diseases and interactions of TRP channels in their pathological progression has been extensively described in the literature. This section briefly reviews TRP channels and diseases to which the cation channel superfamily may contribute or in which it may play a role; it also outlines the current therapeutic options for targeting TRP channels.

By modulating intracellular calcium concentrations or the membrane potential, the cation channels of the TRP family are efficient signal transmitters and are particularly stimulated by vision, olfaction, mechanosensation, and the sense of hearing, as well as by chemical stimuli such as capsaicin or aldehydes. TRP channels are also able to detect changes in their environment, such as osmolarity or changes in the flow of fluids [10,96]. Therefore, TRP channels offer a wide field of research and therapeutical targets not only in sensory, motile, and homeostatic functions, but also in physiological processes and immune response [97,98,99]. TRP channels have additionally been widely discussed regarding their role in cardiovascular diseases [100]. Increasing aldehyde exposure has negative effects on human health, as described before. Aldehydes are described to contribute to cardiovascular disease progression and development, similar to atherosclerosis. These effects are caused by lipid peroxidation, aldehyde-induced oxidative stress, malignant products of oxidative stress, and aldehydic compounds [101]. 

Given the versatility of the activation and inhibition of the TRP channel superfamily members, TRP cation channels associated with group 1 are involved in a multitude of diseases as well. The following paragraph will give a brief overview of the diseases associated with TRP channel group 1 members. TRPC channels have been described to be involved in Parkinson´s disease, as well as in cancer or diseases of the respiratory and pulmonary system, such as asthma and chronic obstructive pulmonary disease (COPD) [9,11,98]. TRPA1 was studied in a murine model of ischemia reperfusion injury and was identified as a regulator of cardiac reperfusion injury by Lu et al. [102]. As the channel is activated by itching and pain, it is also involved in neuropathic pain and inflammatory processes [103], as well as in cold hyperalgesia. There is also a lot of evidence showing that TRPA1 and TRPV play a role in migraines [103,104,105,106]. TRPV channels have also been described to be involved in bladder disease, cancer, and Alzheimer’s disease [9]; diabetes mellitus [60]; in diseases of the cardiovascular, digestive, and respiratory systems [54,107]; as well as in inflammatory processes and immune response regulation [99]. The TRPM cation channels are involved in Alzheimer’s disease, allergy, and stroke, as well as in diabetes mellitus and bladder, breast, and lung cancer [9,108,109]. Mutations in TRPM3 are related to various forms of glaucoma and cataracts [108,110,111], whereas TRPM2 modulates renal fibrosis and inflammatory responses [112,113,114,115]. Members of group 1 TRP channels have been implicated in a variety of diseases to date and the discovery of the novel interactions of TRP channels and their role in pathological conditions is still ongoing.

The group 2-associated TRP channel families were both discovered during research on the associated diseases [100,116,117,118]. The TRPP1 and TRPP2 channels of the TRPP subfamily are widely known to play a role in the development of polycystic kidney disease (PKD) after mutation, leading to the development of various cysts filled with liquid in the kidneys [9,10,119]. The TRPML subfamily is strongly associated with the disease mucolipidosis IV, which is caused by mutations in the TRPML receptor encoding gene MCOLN1 [120]. Mucolopidosis IV is an autosomal recessive neurodegenerative lysosomal storage disorder [121]. 

Given the involvement of TRP channels in various physiological functions and thus in turn also in disease mechanisms, TRP channels may be an attractive therapeutical target. In fact, drugs targeting TRP channels are already on the market for the treatment of TRP-related diseases. The topical application of capsaicin in ointments, for example, is an example of the suitable use of a therapeutic in diabetic neuropathy, osteoarthritis, and post-herpetic neuralgia [122,123,124]. Heat exposure due to sauna or hot tub use is reported to be beneficial for people with coronary risk factors, diabetes mellitus, and vascular endothelial dysfunction [125,126]. In these studies, the positive effect is attributed to the vasodilatory effect, but it is possible that activated TRP channels also play a role in the improvement of these diseases after heat therapy. Garlic, which contains alliin and allicin, is assumed to play a beneficial role in cardiovascular diseases (extensively reviewed in [127]) and also counteracts the adverse effects of the aldehyde-induced pathogenesis of cancer [128]. Aged garlic extracts have been used for their antioxidant properties and reduction in lipid peroxide formation [129,130], although the involvement of TRP channels remains yet to be confirmed. For pain relief, many components targeting TRP channels in clinical trials were extensively reviewed by Brederson et al. The modulation of thermo-sensitive TRP channels is described as a promising opportunity for pain relief. The application of the capsaicin-like resiniferatoxin, resulting in an ablation of sensory neurons, could be a potential therapeutical option for long-term persistent analgesia in patients with localizable chronic pain due to cancer, for example, and it has already been used in clinical trials [131]. Muller et al. also discussed the therapeutic potential of the use of cannabinoid ligands on TRP channels. TRPV1-4, TRPA1, and TRPM8 are known to be receptors modulated by cannabinoid ligands (ionotropic cannabinoid receptors) and are used for the treatment of pain and their analgesic effects [130,132]. 

Therefore, targeting TRP channels to improve several diseases is a long-known therapeutical possibility and is the subject of a broad field of research. However, not only the direct targeting of TRP channels, but also the limitation of aldehydic cellular adducts could be of great potential as an additional therapeutic strategy for improving a multitude of diseases. 

## 6. Modulation of Endogenous Aldehydes to Limit Cellular Adducts: A Potential Therapeutic Strategy 

The TRP channel and aldehyde interaction play major roles in oxidative stress. As a consequence of various diseases or lifestyle choices, such as ethanol or tobacco consumption, excessive lipid peroxidation leads to the accumulation of endogenous aldehydes and aldehydic compounds in toxic amounts, as described earlier in this review. Furthermore, the breakdown of metabolites from arachidonic acids in membranes is a key factor contributing to multiple diseases. In this chapter, we aim to introduce three options modulating the major disease pathophysiologies described in this review. Firstly, we will dissect options to block TRP channels; secondly, we will provide an overview of the strategies used to block HETE formation from arachidonic acid breakdown; thirdly, we will introduce a means to boost aldehyde metabolism. 

The direct inhibition of TRP channels counteracting the effects of channel activation is a potent strategy for the improvement of pain or deleterious cardiovascular effects. A multitude of TRP channels antagonists are extensively reviewed in the study of Brederson et al. [131]. Briefly, the TRPV1 antagonist SB-705498 was declared to be suitable for preclinical studies of pain relief, besides other candidates for TRPV1 antagonization [131,133]. Another possible TRPV1 antagonist is 4-(3-chloro-2-pyridinyl)-N-[4-81,1-dimethylethyl)phenyl]-1-piperazine-carboxamide (BCTC), which has previously been used for the antagonization of TRPV1 in a study of diabetes and in another study on pain relief [60,134]. A protective effect of treatment with BCTC was also shown in a model of murine heart failure [135]. Capsazepine is another TRPV1 antagonist that has been extensively described [136,137,138]. In 2016, Hurt et al. also introduced a peptide, V1-cal, that partially mimics the coiled-coil domain of TRPV1, preventing the interaction of calcineurin, a modulator of the TRPV1 activation state, with the TRPV1 receptor and leading to a protective effect in a model of myocardial reperfusion injury [136]. In addition, the antagonization of TRPV3 in pain or inflammation with small molecules offers a potential therapeutical option (reviewed in [131]). Additionally, the blockage of TRPA1 was described as a potential therapeutical means for pain relief using a selective TRPA1 blocker, A-967079 [139]. TRPA1 antagonization is not only an option for the treatment of patients suffering from chronic pain, but evidence also suggests that it may have pathophysiological involvement in pruritis and inflammation; consequently, it could be a novel basis for further research studies in this area [100,131,140,141,142]. All these TRP antagonists may offer novel strategies of targeting TRP and aldehyde interaction, as the TRP channel is a downstream target of aldehydes, which induce functional changes in the channel. Their interface further plays a key role in physiological processes as well as in disease pathophysiology, as extensively reviewed earlier in Section 4 of this review. For instance, TRP antagonism may abolish calcium influx into cells during aldehyde exposure and may thus be a potent means by which to alleviate adverse consequences. Since aldehydes induce conformational changes via the posttranslational modification of amino acid residues of the channel, which significantly differs from the binding of classic agonists through lock-and-key binding, future research will need to identify which TRP channel antagonists are suitable for blocking aldehyde effects. 

Another option to limit the actions of eicosanoids derived from lipid membrane breakdown is to block the effects of hydroxyeicosatetraenoic acids (HETEs). HETEs are metabolites of the breakdown of arachidonic acids through 15-lipoxygenase (15LOX) or 12-lipooxygenase (12LOX), as described earlier [32]. Recently, data from our research group have shown that endothelium-specific 12/15-lipooxygenase knockout mice were protected against diabetes-induced endothelial dysfunction, similarly to TRPV1 knockout mice [60]. Vice versa, the 12-LOX metabolite 12(S)-HETE, which is increased in diabetic patients, was found to be a potent inductor of mitochondrial dysfunction via affecting mitochondrial TRPV1 function and thus of the impairment of endothelial function [60]. These findings indicate that targeting the endothelial lipid peroxidation metabolism is a potent means by which to alleviate diabetes-induced complications of vascular function. In medical therapy, targeting arachidonic acid metabolism has been used for many years since cyclooxygenase (COX) inhibitors emerged on the market, blocking the COX-mediated breakdown of prostanoids from arachidonic acids [143]. Nonsteroidal anti-inflammatory drugs (NSAIDs), such as aspirin or ibuprofen, are among the most common drugs prescribed worldwide due to their analgetic, antiphlogistic, and antipyretic effects [144,145]. In recent years, an increasing amount of evidence has shown that targeting arachidonic acid metabolism through LOX inhibitors is also a potent means to treat diseases; thus, we will discuss some LOX inhibitors in the following section. For instance, baicalein is a potent enzyme inhibitor of 12- and 15-LOX, exhibiting anti-inflammatory actions in pre-clinical studies [146,147]. As 12-LOX is involved in the modulation of platelet aggregation, ML355, a selective inhibitor of 12-LOX, was successfully used in antiplatelet treatment studies in mice [148]. Furthermore, ML355 administration significantly diminished the formation of 12(S)-HETE in isolated human pancreatic islets and augmented insulin response, suggesting that ML355 exerts beneficial effects in the treatment of diabetes, as marked by an impaired insulin metabolism [149,150,151]. Additionally, Frondanol is a US-patented natural drug that inhibits the 12-LOX pathway. It was found to be effective in the treatment of colon inflammation in a mouse model [152,153]. These previous studies suggest that limiting the formation of HETE by blocking LOX is a potent pharmacological strategy for the treatment of multiple diseases. Further studies will need to determine the effects of LOX inhibition in pre-clinical models as well as in the treatment of patients. Recent evidence from our research group has shown that using a peptide, V1-cal, which mimics a TRPV1 box motif identified as the binding site of 12(S)-HETE, improved mitochondrial function in endothelial cells and subsequently restored endothelium-dependent vasodilation in diabetic mice [154]. We observed similar effects when using the 12-LOX inhibitor baicalein in vitro and ML531 in vivo, as well as from the administration of the TRPV1 channel blocker BCTC [154]. These findings suggest that targeting the interaction between HETEs and the TRP channel is a novel strategy that could be used to specifically preserve vascular function in disease states. 

Reactive aldehydes derived from lipid peroxidation during oxidative stress exert harmful effects on the body, as discussed in this review. The mitochondrial enzyme aldehyde dehydrogenase-2 (ALDH2) not only mediates the oxidation of acetaldehyde to acetic acid in alcohol metabolism but also the detoxification from reactive aldehydes; thus, it mitigates the consequences of aldehyde actions [155,156]. However, if high concentrations of reactive aldehydes such as 4-HNE are present, ALDH2 will be inhibited. leading to the further accumulation of reactive aldehydes and thus amplifying the pro-oxidative cascade [8,157,158].

**Figure 4:**
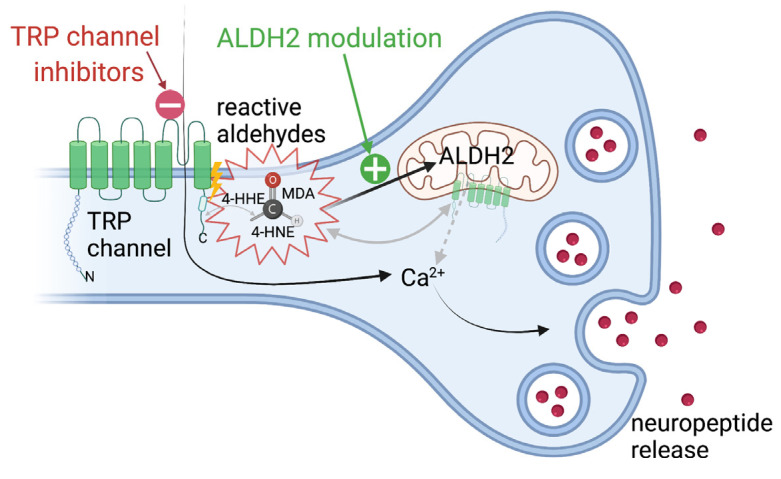
Interaction between reactive aldehydes and TRP channels offering novel therapeutic strategies. Reactive aldehydes are produced upon lipid peroxidation during oxidative stress and specifically modify the C-terminal amino acid residues of TRP channels, which further leads to conformational changes in the channel and subsequent calcium influx into the cell. As evidence pointing towards the TRP channel being located in the mitochondria has been found, aldehyde and TRP channel interaction may also promote calcium release from intracellular stores. AN increase in the level of intracellular calcium triggers the release of pro-inflammatory and pro-algesic neuropeptides, such as substance P and calcitonin gene-related peptide (CGRP). Targeting the TRP channel and aldehyde interaction—either by inhibiting the TRP channel or the upregulation and preconditioning of the mitochondrial enzyme aldehyde dehydrogenase-2 (ALDH2), which mediates detoxification from reactive aldehydes—may specifically target the adverse actions of oxidative stress, driving diseases such as ischemia reperfusion injury, diabetes, neurogenic inflammation, cancer, and neurodegenerative disease. This figure was created with BioRender.com (accessed on 4 September 2021).

ALDH2 may subsequently be a promising novel target in treatment aiming to diminish the effects of endogenous aldehydes on TRP channels in a pro-oxidative setting in diseases driven by toxic endogenous adducts inside the body during diabetes; cardiovascular disease; ischemia reperfusion injury; and neurological pathologies, such as Alzheimer’s disease and Parkinson’s [8,155,159]. ALDH2 is expressed in several cell types and organs, such as the kidney, liver, heart, and brain [160,161]. The interaction of aldehydes and TRP channels and possible strategies for modulating their interface are illustrated in Figure 4.

Interestingly, an ALDH2*2 genetic variant leading to decreased ALDH2 enzymatic activity is present in 40% of the East Asian population, leading to this phenotype being more susceptible to aldehyde toxicity [162].

Recent studies have shown that ALDH2 activity correlates with pain tolerance and modulates nociceptive sensation by the metabolization of aldehydes [163]. Furthermore, the administration of Alda-1 (N-(1,3-benzodioxol-5-ylmethyl)-2,6 di-chloro-benzamide), an activator of ALDH2, protects against ischemia reperfusion injury and ALDH2 upregulation, leading to aldehyde clearance that may thus prove beneficial in myocardial ischemia-reperfusion, coronary bypass surgery, or the reperfusion of an organ transplant [164]. In endometriosis, the TRPA1 and TRPV4 channels are up-regulated and reactive aldehydes, such as 4-HNE, accumulate in the peritoneal fluid of affected women, pointing towards a potential interaction between aldehydes and TRP channels promoting the disease pathophysiology [165,166,167]. Increasing the ALDH2 activity through Alda-1 was found to diminish endometriosis lesion formation and further reduce pain in rodents [168]. Modulating the ALDH2 activity may thus also benefit patients suffering from endometriosis. In their studies, Fu et al. demonstrated that treatment with Alda-1 was able to improve brain injury due to the activation of ALDH2 and reduction in reactive aldehyde accumulation in a rodent model, pointing towards its beneficial effects in neurodegenerative disease [169]. Additionally, Zhong et al. studied the reduction of aldehydes with Alda-1 treatment in alcoholic steatosis, resulting in improved alcoholic liver disease in mice [170]. 

Besides the ability of Alda-1 to induce ALDH2 expression, another option to upregulate aldehyde clearance is to utilize various compounds that activate ALDH2. For instance, isoflurane could be used for preconditioning, as ALDH2 is triggered by the cardiovascular protection mechanisms after isoflurane exposure, which may impact the anesthesia of patients at high risk of exposure to oxidative stress during organ transplant or on-pump cardiac bypass surgery. Another preconditioning agent could be ethanol, as ALDH2 plays a role in alcohol metabolism, as described before [8]. Finally, these data reveal that the upregulation or preconditioning of ALDH2 may be a potent means by which to amplify detoxification from reactive aldehydes and further diminish their action on TRP channels. 

To conclude, there is a strong basis for novel translational research studies dissecting the topics discussed in this review and providing novel therapeutic strategies for a multitude of diseases. 

## 7. Conclusions and Future Perspectives

Evidence suggests that targeting reactive aldehydes is a potent means to alleviate the adverse consequences of an increased endogenous aldehyde load. Endogenous reactive aldehydes are produced by the lipid peroxidation of polyunsaturated fatty acids and induce the activation of TRP channels by the covalent modification of specific intracellularly located amino acid residues, thus promoting conformational changes. The induction of channel opening by electrophilic aldehydes significantly differs from the binding of classic agonists, which use a lock-and-key binding mechanism, which shows that TRP channels play a key role in sensing oxidative stress beyond mediating nociception (their original function, which was discovered first). Interaction with aldehydes leads to intracellular calcium influx, triggering the release of pro-inflammatory and pro-analgesic mediators. Since reactive aldehydes are elevated during acute oxidative insults, such as ischemia reperfusion injury, as well as in chronic diseases such as diabetes, neurogenic inflammation, cancer, and neurodegenerative disease, it could be suggested that they are far more than merely a by-product of oxidative stress. As discussed in this review, reactive aldehydes play a crucial role in disease promotion. They may thus aid in the risk stratification of patients and may further display a novel target in treatment. Aldehyde dehydrogenase-2 (ALDH2) mediates detoxification from reactive aldehydes and its upregulation and preconditioning may thus play a key role in therapy aiming for the clearance of reactive aldehydes. Recent studies have revealed that the induction of ALDH2 by Alda-1 protects against ischemia reperfusion injury and ameliorates lesion formation in endometriosis and neurodegenerative disease. 

Future studies aiming to understand TRP channels as biosensors for reactive aldehydes and target their interface will certainly provide a more complete picture, enabling us to understand the associated disease pathophysiology. Since the body is also exposed to exogenous aldehydes—for example, via tobacco smoke, air pollution, or alcoholic beverages, which have been known to impair health for years—this broadens the impact of aldehyde action on the human body. Targeting this interaction—either by the blockage of TRP channels or the reduction of reactive aldehydes—may usher in a new era of disease treatment options and health protection.
